# Obstetric Ultrasound Screening in Lebanon for Fetal Diagnosis and Associated Factors of Congenital Abnormalities

**DOI:** 10.3390/children12081076

**Published:** 2025-08-15

**Authors:** Rita Chebl, Ingrid Nader, Michel Saba, Cecile Z. Attieh, Ogarite Kattan, Lea Nohra, Anna-Maria A. Henaine, Sarah El Khoury, Malek N. Nassar, Pierre Nakhel, Béchara El Asmar, Mirna N. Chahine

**Affiliations:** 1 Faculty of Medical Sciences, Lebanese University, Hadath P.O. Box 3, Lebanon; r.chebl@st.ul.edu.lb (R.C.); i.nader.1@st.ul.edu.lb (I.N.); saba.michel90@gmail.com (M.S.); o.kattan@st.ul.edu.lb (O.K.); nohra.lea@st.ul.edu.lb (L.N.); pnakhel@gmail.com (P.N.); 2 Faculty of Medicine &Medical Sciences, University of Balamand, Koura Campus, Tripoli P.O. Box 100, Lebanon; cecile.attieh@std.balamand.edu.lb; 3 Faculty of Pharmacy, Lebanese University, Hadath P.O. Box 3, Lebanon; a.henaine@ul.edu.lb; 4 Lebanese Association of the Knights of Malta, Order of Malta Lebanon, Vanlian Bldg, 6th Fl. City Rama Str. Dekwaneh, Beirut P.O. Box 11-4286, Lebanon; elkhourysarah@yahoo.com (S.E.K.); drmaleknassar@yahoo.fr (M.N.N.); bechara.elasmar@hdf.usj.edu.lb (B.E.A.); 5 Department of Obstetrics and Reproduction, Hotel-Dieu de France Hospital, Beirut P.O. Box 11-5190, Lebanon; 6 Department of Obstetrics & Gynecology, Faculty of Medical Sciences, Lebanese University, Hadath P.O. Box 3, Lebanon; 7 Faculty of Medicine, Saint Joseph University, Beirut P.O. Box 17-5208, Lebanon; 8 Department of Cardiology, Hotel-Dieu de France Hospital, Beirut P.O. Box 11-5190, Lebanon; 9 Department of Biology, Faculty of Arts & Sciences, University of Balamand, Dekwaneh Campus, Sin-el-Fil P.O. Box 55251, Lebanon; 10 Foundation-Medical Research Institutes (F-MRI^®^), Beirut P.O. Box 166378, Lebanon; 11 Foundation-Medical Research Institutes (F-MRI^®^), 1211 Geneva, Switzerland

**Keywords:** obstetric ultrasound, screening, congenital abnormalities, Lebanon

## Abstract

**Highlights:**

**What are the main findings?**

- In a multicenter Lebanese cohort, 13.1% of pregnancies screened via second-trimester ultrasound had congenital abnormalities, including 8.5% growth abnormalities and 10.1% morphological malformations.
- Several maternal and clinical factors—including advanced maternal age, parity, obstetric complications, maternal anxiety, and a prior history of anomalies—were significantly associated with adverse fetal outcomes.

**What is the implication of the main finding?**

- The results highlight the need to integrate systematic second-trimester prenatal ultrasound screening into national antenatal care protocols, particularly in settings with limited healthcare resources.
- Strengthening access to timely screening, provider training, and structured follow-up could improve early detection, intervention, and ultimately maternal and neonatal health outcomes in Lebanon.

**Abstract:**

**Background and Objectives**: Congenital abnormalities are a leading cause of neonatal morbidity and mortality and are frequently detectable through prenatal ultrasound. While widely implemented in high-income countries, such screening remains inconsistently applied in low- and middle-income regions. This study aimed to estimate the prevalence of congenital abnormalities identified via prenatal ultrasound in Lebanon and to explore associated maternal, obstetric, and psychosocial factors. **Methods**: A multicenter retrospective observational study, supplemented by follow-up interviews, was conducted in five Order of Malta medical centers. Pregnant women in their second trimester underwent an obstetric ultrasound, and data were collected through structured questionnaires and follow-up phone interviews. Variables included maternal demographics, obstetric history, anxiety levels (GAD-7 scores), and ultrasound findings. **Results**: A total of 426 pregnant women were enrolled (mean age: 28.8 ± 5.9 years). The overall prevalence of congenital abnormalities was 13.1%. Growth abnormalities were observed in 8.5% of fetuses and were significantly associated with obstetric complications and the presence of multiple abnormalities. Morphological malformations were found in 10.1% of cases and were more common among women of advanced maternal age, those with a history of anomalies, and those reporting elevated anxiety scores. Combined abnormalities, as well as growth and morphological malformations, were significantly associated with higher parity, prior anomalies, and current pregnancy complications. **Conclusions**: Prenatal ultrasound is essential for early detection of congenital abnormalities, facilitating timely intervention and improved neonatal outcomes. These findings emphasize the need to integrate systematic screening into prenatal care in Lebanon and for ongoing research to identify context-specific risk factors.

## 1. Introduction

Congenital abnormalities, or birth defects, represent a major global health concern due to their significant contribution to infant morbidity and mortality. These conditions can be detected prenatally, at birth, or later in life, and span a wide spectrum—from mild, treatable anomalies to severe, life-threatening malformations [1,2]. As other causes of mortality among children under five have declined, congenital anomalies have emerged as a growing proportion of under-five deaths, accounting for 4% to 30% of such deaths globally, depending on the region [3].

According to the World Health Organization (WHO), approximately 6% of infants worldwide are born with congenital disorders [2], with the highest burden falling on low- and middle-income countries (LMICs), where an estimated 90% of severe congenital malformations occur [4]. Prevalence rates vary widely between countries: 4.24% in Pakistan [5], 0.6% in Barbados [6], 1.85% in India [7], 29% in Tanzania [8], and 2.5% to 5.95% in various studies from Ethiopia [9,10,11], as well as 2.8% in Ghana [12] and 1.3% in Qatar [13]. The most commonly affected systems include the central nervous, cardiovascular, and musculoskeletal systems [9,14].

The etiology of congenital abnormalities is multifactorial, involving genetic predispositions, environmental exposures, infectious agents, nutritional deficiencies, and socio-economic determinants [2,4]. Given this complexity, studies investigating congenital anomalies must consider a wide range of demographic and contextual factors to generate meaningful insights.

A key advancement in the early detection of such abnormalities has been the introduction of prenatal obstetric ultrasound. This non-invasive technique has become essential in modern obstetric care, enabling early identification of fetal growth restrictions, chromosomal anomalies, and structural malformations throughout different stages of pregnancy [15,16,17]. International studies highlight its value: in England, for example, Gardosi et al. found a strong link between undetected fetal growth restriction and stillbirths, while Eleftheriades et al. demonstrated that up to 45% of congenital heart defects could be identified through ultrasound screening [18,19].

In Lebanon, however, the application of prenatal ultrasound as a routine screening tool faces numerous obstacles. The country’s ongoing economic crisis, political instability, and the strain of a prolonged refugee crisis have compromised the healthcare infrastructure, particularly maternal and child health services [20,21]. These challenges disproportionately affect vulnerable populations and hinder access to essential antenatal screenings, including ultrasound examinations. To date, limited research has been conducted on congenital anomalies in Lebanon. Bittar et al. (1995) reported a prevalence of 16.5 anomalies per 1000 births, with skeletal anomalies being the most common [22], while a more recent study by Snaifer et al. (2021) among a refugee population reported growth abnormalities in 20% of pregnancies and morphological malformations in 12% [23]. However, these studies are either outdated or narrowly focused, underscoring the need for broader, more recent investigations.

In light of these gaps, the present study aims to estimate the prevalence of congenital abnormalities identified through obstetric ultrasound screening in Lebanon and to examine their association with maternal, obstetric, and psychosocial factors. Additionally, this study explores how these abnormalities impact pregnancy outcomes across a more representative and diverse Lebanese population.

## 2. Methods

**Figure 1:**
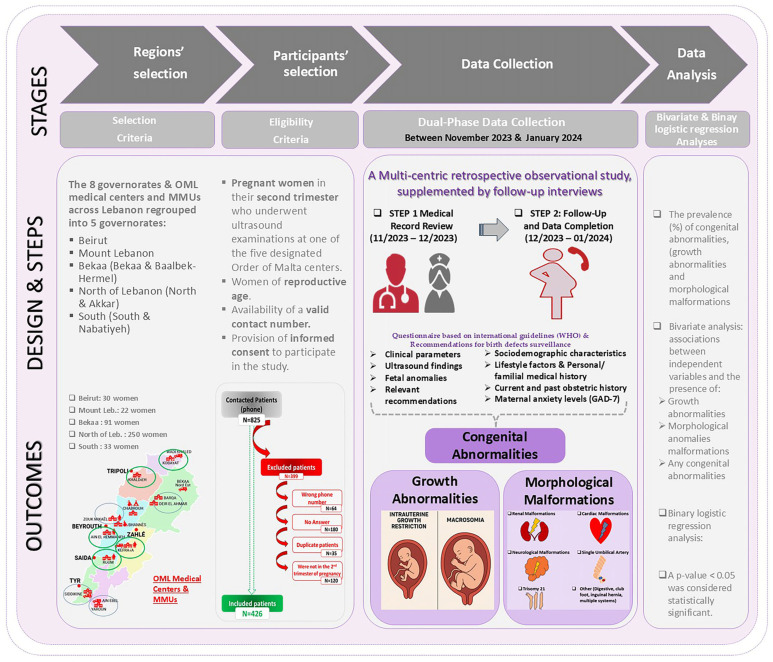
The study flow including the contents, participant selection, methods, and dual-phase data collection. On the map of Lebanon, green circles indicate the five OML medical centers where ultrasound examination was conducted, while all circles represent the geographical distribution of participants’ residences. OML: Order of Malta; MMUs: Mobile Medical Units; WHO: World Health Organization.

The study flow is represented in Figure 1.

### 2.1. Study Design and Participants

This multi-centric retrospective observational study, supplemented by follow-up interviews, was conducted among pregnant women in their second trimester who underwent obstetric ultrasound examinations at five of the eleven Order of Malta medical centers across various Lebanese governorates: Kobayat (Akkar), Khaldieh (North), Ain El Remmaneh (Beirut), Kefraya (Bekaa), and Roum (South). These centers were strategically selected to ensure geographic and sociodemographic diversity, as well as to reflect a broad cross-section of maternal populations in both rural and peri-urban settings. Selection was based on key criteria including wide patient catchment areas, availability of standardized prenatal gyneco-obstetric services (including ultrasound screening), and sufficient infrastructures, such as trained personnel, consistent antenatal care follow-up, and electronic record systems, needed to ensure reliable data extraction and support retrospective analysis. Importantly, although data collection was limited to these five centers, the study population included pregnant women residing in the eight Lebanese governorates, thereby enhancing the national representativeness of the sample.

The second trimester was specifically chosen as it represents the optimal window for detecting structural congenital abnormalities. During this period (typically between 18 and 24 weeks of gestation), the fetal anatomy is sufficiently developed to allow for reliable identification of growth abnormalities and morphological malformations in accordance with international guidelines [15]. Moreover, in the Lebanese context, the second-trimester ultrasound is the most routinely available and standardized screening tool across maternal care facilities.

Data were collected from patient medical records and ultrasound reports. To address gaps in archived data, follow-up phone interviews were conducted with patients to obtain missing information.

#### 2.1.1. Inclusion Criteria

Participants were included based on the following criteria:

- Pregnant women in their second trimester who underwent ultrasound examinations at one of the five designated Order of Malta centers.
- Women of reproductive age.
- Availability of a valid contact number.
- Provision of informed consent to participate in the study.

#### 2.1.2. Exclusion Criteria

The exclusion criteria were as follows:

- Pregnant women who underwent ultrasounds at non-participating medical facilities.
- Those with only first- or third-trimester ultrasound scans.
- Invalid or incorrect contact information.
- Refusal to provide consent or participate in follow-up.

#### 2.1.3. Sample Size

The sample size was determined using Slovin’s formula:

n = N/(1 + Ne^2^)

where

- n is the required sample size,
- N is the estimated annual number of pregnant women in Lebanon (approximately 70,000),
- e is the margin of error (set at 0.05).

Applying the formula, a minimum of 400 participants was required to achieve a representative sample of the Lebanese pregnant population.

### 2.2. Data Collection

#### 2.2.1. Dual-Phase Data Collection: Medical Record Review and Follow-Up Interviews

Data collection was conducted in two sequential phases:

1. Medical Record Review (10 November–10 December 2023):

Medical records of pregnant women who underwent second-trimester ultrasound examinations (Voluson S6 ultrasound system (GE Healthcare, Seoul, Republic of Korea)) at Order of Malta health centers were systematically reviewed. These examinations were primarily conducted by obstetrician–gynecologists, mainly Dr Malek Nassar, with the assistance of trained midwives. Clinical parameters, ultrasound findings, and any detected fetal anomalies were thoroughly documented. Relevant recommendations made by healthcare providers were also recorded (Figure 1).

2. Follow-Up and Data Completion (14 December 2023–3 January 2024):

To address missing or incomplete data, follow-up phone interviews were conducted with the participants. A structured questionnaire was used to gather information across five primary domains based on the participants’ recollection at the time of the ultrasound:

- Sociodemographic characteristics.
- Lifestyle factors and personal/familial medical history.
- Current and past obstetric history.
- Maternal anxiety levels.
- Ultrasound findings and subsequent clinical recommendations (Figure 1).

The questionnaire incorporated variables adapted from Snaifer et al. (2021) [23] and Abebe et al., 2021 [24], in addition to the Generalized Anxiety Disorder scale (GAD-7) [25]. The questionnaire was developed by adapting validated instruments to ensure content validity and reliability. The GAD-7 scale is a widely validated tool for assessing maternal anxiety [25]. Other sections—including sociodemographic, lifestyle, personal and familial medical history, and obstetric data—were adapted from structured data collection tools used in prior studies with established methodologies for congenital anomaly research [23,24]. Additionally, the questionnaire design was based on international guidelines and recommendations for birth defects surveillance and data collection, such as those by DeSilva et al. (2016) [1], World Health Organization reports on birth defects [2,3,4], and related epidemiological studies [9,10,14]. Prior to use, the questionnaire was pilot-tested for clarity and comprehensibility in the target population. Phone interviews were conducted by trained medical doctors following a structured script to maintain consistency and minimize interviewer bias.

#### 2.2.2. Data Integration and Consistency Measures

This study was conducted as a multicenter retrospective observational study supplemented by follow-up interviews. Clinical data were initially extracted from medical records and ultrasound reports during the second trimester. However, certain non-clinical variables—particularly sociodemographic characteristics, lifestyle behaviors, obstetric history, and maternal anxiety—were not consistently documented. To address this, standardized follow-up phone interviews were conducted to retrospectively collect missing information. These interviews were structured to align temporally with the second-trimester period and were designed to minimize recall bias by clearly anchoring participant responses to the time of the ultrasound. Interviewers received training to ensure consistent administration of the questionnaire, and any inconsistencies were flagged and clarified through cross-referencing with the available clinical documentation.

### 2.3. Definitions and Classification of Fetal Abnormalities

Fetal abnormalities detected by prenatal ultrasound were classified into two main categories to ensure clarity and consistency in data analysis.

“Growth abnormalities” refer to deviations from normal fetal growth patterns, including intrauterine growth restriction (IUGR) and macrosomia, characterized by fetal size parameters that fall below or exceed established gestational age norms. These definitions align with the clinical and epidemiological guidelines provided by Mayer and Joseph (2013) [26], as well as the World Health Organization’s classifications of fetal growth disorders [2,3].

“Morphological malformations” comprise structural malformations affecting one or more fetal organ systems, such as congenital heart defects, neural tube defects, renal malformations, and other major congenital anomalies. This classification follows internationally accepted case definitions and guidelines for congenital anomaly surveillance as outlined by DeSilva et al. (2016) [1], the World Health Organization (2023) [3], and previous epidemiological studies [6,9,22].

By distinctly categorizing growth abnormalities and morphological malformations, this study aimed to reduce overlap and improve the specificity of findings, thus addressing common ambiguities highlighted in congenital anomaly research.

### 2.4. Statistical Analysis

All statistical analyses were conducted using IBM SPSS Statistics, version 27.

Descriptive statistics were used to summarize the data:

- Nominal variables were expressed as frequencies and percentages.
- Continuous variables were presented as means and standard deviations.
- The prevalence of congenital abnormalities, including growth abnormalities and morphological malformations, was calculated and reported in percentage terms.

Bivariate analysis was conducted to examine associations between independent variables and the presence of the following:

- Growth abnormalities (Yes/No).
- Morphological malformations (Yes/No).
- Any congenital abnormalities (Yes/No).

The statistical tests used included the Chi-square test, Fisher’s exact test, and Student’s *t*-test where appropriate. A *p*-value < 0.05 was considered statistically significant.

Anxiety was assessed using the Generalized Anxiety Disorder scale (GAD-7). GAD scores were initially analyzed both as continuous variables and in four clinically defined categories: minimal anxiety (GAD 0–4); mild anxiety (GAD 5–9); moderate anxiety (GAD 10–14); and severe anxiety (GAD 15–21). Given the observed association between congenital abnormalities and moderate anxiety, an additional dichotomous analysis was conducted, categorizing GAD scores as low anxiety (GAD 0–9) and high anxiety (GAD ≥ 10). This simplified classification allowed for improved interpretability and was analyzed using a 2 × 2 contingency table, with odds ratios and 95% confidence intervals calculated to quantify the strength of association.

Binary logistic regression was employed to identify risk factors independently associated with each of the three anomaly categories set as variables, such as growth abnormalities, morphological malformations, and congenital abnormalities. Variables with significant associations in the bivariate analysis were included in the multivariate model.

### 2.5. Ethical Consideration

Ethical approval for this study was granted by the Ethics Committee of Lebanese Hospital Geitaoui (Approval #2023-IRB-019; dated 3 November 2023). This study was conducted in accordance with the Declaration of Helsinki guidelines for research involving human subjects.

Prior to participation, all eligible women were informed of the study objectives and procedures, and their rights. Informed consent was obtained from each participant, which included the following points:

- Participation was entirely voluntary, with the right to withdraw at any time without consequence.
- Confidentiality and anonymity of all personal and medical data were guaranteed.
- Consent was also sought for the potential future use of anonymized data in both published and unpublished research.

Given the study’s dual data collection approach—a retrospective review of clinical records supplemented by follow-up phone interviews—special attention was paid to ensuring ethical compliance for both phases. Participants were informed not only about the use of their existing medical data but also about the purpose and procedures of the follow-up interviews, including the voluntary nature of participation and their right to withdraw without any impact on their care. Contact information was securely stored and used solely for data completion. All data were anonymized prior to analysis to protect participant identity and privacy.

## 3. Results

### 3.1. Demographic and Maternal Health Characteristics

#### 3.1.1. Demographic Profile

A total of 825 medical records of pregnant women who underwent second-trimester ultrasound examinations (Voluson S6 ultrasound system) at Order of Malta health centers were initially reviewed. Subsequently, follow-up phone interviews were conducted with these individuals. Figure 1 presents the study flowchart, detailing the inclusion and exclusion process, along with the reasons for participant exclusion. A total of 399 pregnant women were excluded due to incorrect contact information (a wrong or invalid phone number), lack of response, duplicate entries, or not meeting the second-trimester pregnancy criteria. Following the completion of the interviews, 426 pregnant women met the inclusion criteria and were ultimately included in this study.

The majority were between 18 and 30 years of age (n = 252; 59.2%), followed by women aged 31 to 40 years (n = 155; 36.4%). A smaller proportion were younger than 18 years (n = 5; 1.2%) or older than 40 years (n = 14; 3.3%). The mean maternal age was 28.8 years (SD = 5.93), with a range from 14 to 49 years.

Educational attainment varied across women: 2.8% were illiterate (n = 12), 20.7% had completed primary education (n = 88), 17.6% had complementary-level education (n = 75), and 15.0% had secondary education (n = 64). Notably, 41.3% were university graduates (n = 176), and 2.6% had pursued post-graduate studies (n = 11). Additional demographic details are presented in Table 1.

#### 3.1.2. Lifestyle Habits

The majority of participants reported non-smoking status (n = 317; 74.4%), while 25.6% (n = 109) identified as smokers. Regarding alcohol consumption, 88.3% (n = 376) reported no history of alcohol intake. These lifestyle behaviors, summarized in Table 1, provide context for interpreting potential maternal risk factors influencing fetal development and congenital abnormalities.

#### 3.1.3. Comorbidities and Obstetric History

Among the fetuses, 51.4% were male (n = 219) and 45.1% were female (n = 192), while 0.7% (n = 3) had undetermined sex. Twin pregnancies accounted for 2.3% (n = 10), and triplet pregnancies for 0.5% (n = 2).

A substantial proportion of participants reported a history of abortion (n = 166; 39.0%), with an average of 1.62 abortions per affected participant (SD = 0.96; range: 1–7). The mean gravidity across the cohort was 3.02 (SD = 2.09; range: 1–22), and the mean parity was 2.26 (SD = 1.35; range: 0–9). Full details of obstetric and medical history are detailed in Table 1.

#### 3.1.4. History of Congenital Anomalies

A previous history of congenital anomalies was reported by 41 participants (9.6%), while the remaining 385 women (90.4%) indicated no such history.

#### 3.1.5. Anxiety Assessment

The Generalized Anxiety Disorder 7-item (GAD-7) scale revealed a mean score of 10.95 out of 21 (SD = 6.39), suggesting a moderate level of anxiety symptoms across the study cohort. Based on the GAD-7 classification, 15.0% of participants experienced minimal anxiety, 31.0% had mild anxiety, another 31.0% showed moderate symptoms, and 23.0% reported severe anxiety levels.

### 3.2. Pregnancy Complications and Follow-Up

#### 3.2.1. Fetal Growth Abnormalities and Morphological Malformations

+ Prevalence

Among the 426 pregnancies evaluated, 36 cases (8.5%) exhibited growth abnormalities, with the majority (n = 390; 91.5%) demonstrating normal growth patterns. Intrauterine growth restriction (IUGR) was the predominant growth disorder, accounting for 83.9% (n = 26) of the abnormal growth cases.

Morphological malformations were identified in 43 cases (10.1%), while 383 pregnancies (89.9%) showed no structural abnormalities.

+ Multiple Abnormalities (Cases with >1 Abnormalities)

A total of 24 pregnancies (5.6%) were found to have more than one type of abnormality, whereas 402 cases (94.4%) involved no concurrent abnormalities.

#### 3.2.2. Amniotic Fluid and Other Obstetric Complications

+ Prevalence of Amniotic and Obstetric Issues

**Figure 2:**
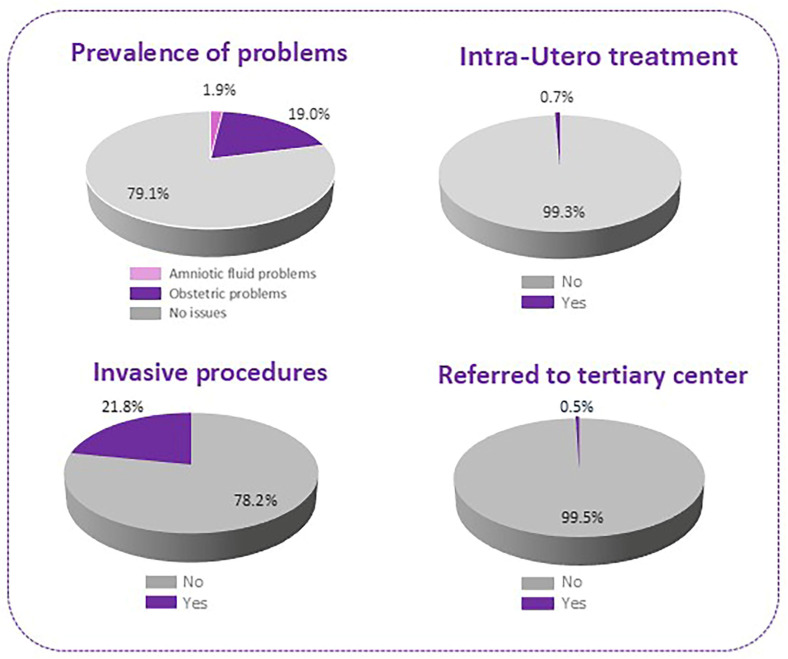
Obstetric complications and their treatment.

Amniotic fluid anomalies were relatively rare, occurring in only eight cases (1.9%). In contrast, obstetrical complications were reported in 81 participants (19.0%). Details are illustrated in Figure 2.

+ Intrauterine Treatment

Of the 426 pregnancies screened, only three cases (0.7%) required intrauterine treatment, while the vast majority (n = 423; 99.3%) did not undergo such interventions (Figure 2).

+ Invasive Procedures

Invasive procedures were performed in 93 participants (21.8%), whereas 333 women (78.2%) did not undergo any invasive diagnostic or therapeutic intervention (Figure 2).

+ Referral to Tertiary Care Centers

Referral to a tertiary care center was documented in only two cases (0.5%), with the overwhelming majority (n = 424; 99.5%) managed within the existing primary care infrastructure (Figure 2).

#### 3.2.3. Follow-Up During and After Pregnancy

+ Antenatal Follow-Up

Antenatal follow-up was considered necessary in 76 cases (17.8%). The most frequently required interventions included serial echocardiography at short intervals (18.4%, n = 14), high-risk pregnancy management due to potential miscarriage (17.1%, n = 13), and close monitoring of fetal growth (10.5%, n = 8).

+ Postnatal Follow-Up

Postnatal follow-up was indicated in 38 cases (8.9%). Among these, the majority required follow-up after emergency cesarean section (78.9%, n = 30). Additional needs included neonatal sepsis surveillance (15.8%, n = 6), management of maternal infections (31.6%, n = 12), and postpartum hemorrhage monitoring (13.2%, n = 5).

### 3.3. Factors Associated with Fetal Growth Abnormalities, Morphological Malformations, and Congenital Anomalies

#### 3.3.1. Factors Associated with Growth Abnormalities

+ Demographic Characteristics

No statistically significant associations were found between growth abnormalities and maternal age (*p* = 0.349), nationality (*p* = 0.540), religion (*p* = 0.958), educational attainment (*p* = 0.452), employment status (*p* = 0.774), household income (*p* = 0.081), living governorate (*p* = 0.099), type of living arrangement (*p* = 0.296), physical activity levels (*p* = 0.777), or maternal blood type (*p* = 0.838).

However, a significant relationship was observed between marital status and growth abnormalities (*p* < 0.001). In addition, the type of residence was significantly associated with abnormal fetal growth (*p* = 0.028), with women living in apartments showing a lower incidence of growth abnormalities (66.7%) compared to those residing in other types of housing (33.3%).

+ Comorbid Conditions

No significant associations were observed between growth abnormalities and smoking status (*p* = 0.629), the average number of cigarettes smoked per day (*p* = 0.119), shisha use per week (*p* = 0.656), or the smoking duration (*p* = 0.428). Likewise, alcohol consumption and its frequency did not significantly correlate with the presence of growth abnormalities (*p* = 0.903 and *p* = 0.911, respectively).

Conversely, hypertension was significantly associated with fetal growth abnormalities (*p* = 0.003). Among women with growth abnormalities, 5.6% were hypertensive, compared to only 0.5% among those without abnormalities (Table 2).

+ Gynecological and Obstetric History

No significant associations were found between growth abnormalities and a history of abortion (*p* = 0.729), stillbirth (*p* = 0.912), gestational age (*p* = 0.663), or gravidity (*p* = 0.177).

Parity, however, was significantly associated with the occurrence of growth abnormalities (*p* = 0.044), indicating that an increased number of prior live births may be linked to a higher risk of growth-related complications (Table 2).

+ Growth Abnormalities and Anxiety

This study evaluated the relationship between anxiety levels, as measured by the Generalized Anxiety Disorder (GAD-7) scale, and the occurrence of growth abnormalities in pregnancies.

The analysis did not show a significant overall correlation between GAD-7 scores and the presence of growth abnormalities (*p* = 0.568). When looking at specific anxiety levels, there was a noticeable distribution across the groups. Among patients with growth abnormalities, 8.3% reported minimal anxiety (GAD 0–4), 27.8% reported mild anxiety (GAD 5–9), and 13.9% reported severe anxiety (GAD 15–21). In contrast, among those without growth abnormalities, the corresponding proportions were 15.6%, 31.3%, and 23.8%, respectively. Specifically, patients with moderate anxiety (GAD 10–14) comprised the highest percentage (50.0%) of those with growth abnormalities (versus 29.2% of those without growth abnormalities), but this did not reach statistical significance (*p* = 0.063). It suggests a trend that may warrant further investigation.

+ Growth Abnormalities and Other Factors

No statistically significant association was found between amniotic fluid abnormalities and the presence of growth abnormalities (*p* = 0.119), although the percentage of cases was higher among those with growth abnormalities (5.6%) compared to those without (2.0%). Specifically, 2 out of 36 patients with growth abnormalities and 8 out of 400 without such abnormalities had amniotic fluid disorders. The odds of amniotic fluid abnormalities were estimated to be nearly 2.8 times higher in the group with growth abnormalities (OR = 2.78; 95% CI: 0.58–13.3), suggesting a potential trend, despite the lack of statistical significance. In contrast, obstetric complications were significantly associated with growth abnormalities (*p* = 0.007). Specifically, 27.8% of pregnancies with growth abnormalities also experienced obstetric problems, compared to only 11.8% of those without such abnormalities.

+ Binary Logistic Analysis for the Risk Factors of Growth Abnormalities

A binary logistic regression was conducted to identify significant predictors of fetal growth abnormalities, including obstetric complications and the presence of multiple abnormalities (Table 3).

The presence of obstetric problems—such as a thin lower uterine segment, isthmocele, preterm contractions during examination, uterine artery notch, and ovarian cysts—was significantly associated with an increased risk of growth abnormalities (OR = 4.254; 95% CI: 1.830–9.891; *p* = 0.001), indicating that such conditions elevate the likelihood of impaired fetal development. Additionally, pregnancies presenting with more than one abnormality were nearly 13.7 times more likely to involve growth abnormalities compared to those without multiple findings (OR = 13.721; 95% CI: 5.085–37.022; *p* < 0.001), representing the most influential predictor in the model (Table 3).

These findings underscore the importance of identifying and managing obstetric complications and coexisting anomalies during pregnancy to mitigate the risk of fetal growth abnormalities.

#### 3.3.2. Factors Associated with Morphological Malformations

+ Morphological Malformations and Demographics

A significant association was found between maternal age and the presence of morphological malformations. The mean age was notably higher among women with malformations (33.19 years) compared to those without (28.52 years), suggesting that advanced maternal age may be a potential risk factor.

Additionally, living conditions were significantly associated with the incidence of malformations (*p* = 0.005). Women residing with others or in non-apartment housing types (e.g., shelters or alternative arrangements) demonstrated a higher prevalence of morphological anomalies.

In contrast, there were no statistically significant associations between morphological malformations and monthly income (*p* = 0.370), physical activity level (*p* = 0.900), or maternal blood type (*p* = 0.843).

+ Morphological Malformations, Lifestyle Habits, and Comorbidities

Neither alcohol consumption nor its frequency was significantly associated with morphological malformations (*p* = 0.351 and *p* = 0.221, respectively). Similarly, no significant correlations were observed with hypertension (*p* = 0.608) or diabetes (*p* = 0.496).

The fetal phenotype (male, female, twins, triplet, or unknown) also showed no statistically significant relationship with the occurrence of malformations (*p* = 0.253).

Furthermore, the mean weight, height, and BMI of participants did not significantly differ between groups with and without morphological malformations (*p* > 0.05), suggesting these anthropometric factors are not key predictors.

+ Morphological Malformations and Gyneco-Obstetrical Factors

A history of stillbirths was significantly linked to the occurrence of morphological malformations (*p* = 0.038). Similarly, a history of abortion also showed a significant association with morphological malformations. Among women with such malformations, 65.4% reported a history of abortion compared to 37.3% among those without (*p* = 0.004), indicating a possible correlation. However, the actual number of previous abortions did not differ significantly between the two groups (*p* = 0.363).

The mean gestational age was slightly lower among pregnancies with morphological malformations (22.61 weeks) compared to those without (23.76 weeks), with this difference reaching statistical significance (*p* = 0.041).

Both gravidity and parity were significantly higher among those with malformations (*p* < 0.001 for both), suggesting that an increased number of pregnancies and births may elevate the risk.

However, no significant associations were observed with consanguinity, COVID-19 vaccination status, infertility, or exposure to infections or radiation during the first trimester (Table 4).

+ Morphological Malformations and Anxiety

Patients with morphological malformations reported significantly higher anxiety levels. The mean GAD-7 score was 13.96 (SD = 4.10) in the malformation group compared to 10.75 (SD = 6.46) in the non-malformation group, with a statistically significant difference (*p* = 0.013). These findings suggest a link between higher maternal anxiety and the presence of fetal morphological malformations (Table 5). This suggests that patients with morphological malformations tend to experience higher levels of anxiety on average.

+ Morphological Malformations and Other Factors

The occurrence of amniotic fluid disorders (e.g., oligohydramnios, polyhydramnios, or anhydramnios (absence of amniotic fluid)) was higher among patients with malformations (7.7%) compared to those without malformations (2.0%). Although this difference was not statistically significant (*p* = 0.119), the odds of amniotic fluid disorders were approximately four times greater in the malformation group (OR = 4.08; 95% CI: 0.74–22.5), suggesting a potential association that merits further investigation. Obstetric complications—such as a thin lower uterine segment, isthmocele, preterm contractions, uterine artery notch, and ovarian cysts—were evenly distributed between the groups (*p* = 1.000). Similarly, intrauterine treatments (*p* = 1.000) and invasive procedures (*p* = 0.086) were infrequently required in both groups. Referrals to tertiary care centers were rare among both groups, with no significant difference in occurrence (*p* = 0.118). However, there was a slightly higher, yet not statistically significant, percentage of patients with malformations being referred to tertiary care centers.

The presence of more than one abnormality was significantly associated with morphological malformations (*p* < 0.001). While only 3.3% of patients without malformations had more than one abnormality, this proportion rose substantially to 42.3% among those with malformations. This finding underscores the complex nature of morphological malformations, which accompany multiple abnormalities. (Table 5).

+ Binary Logistic Analysis of Risk Factors for Morphological Malformations

Binary logistic regression identified several significant predictors of morphological malformations (Table 6).

Maternal age was found to be a statistically significant factor, with each additional year increasing the odds of morphological malformations by 14.1% (OR = 1.141; 95% CI: 1.046–1.245; *p* = 0.003), indicating that advancing maternal age is associated with a higher risk. Although this increase per year may seem modest, it is clinically relevant as the cumulative effect over multiple years substantially elevates the risk, highlighting maternal age as an important consideration in prenatal risk assessment. A previous history of congenital anomalies remained a strong independent predictor, increasing the odds nearly sevenfold (OR = 6.982; 95% CI: 2.064–23.616; *p* = 0.002) and underscoring the influence of persistent genetic or environmental risk factors. Additionally, elevated Generalized Anxiety Disorder (GAD-7) scores were significantly associated with the risk of morphological malformations. For each unit increase in GAD-7 score, the odds increased by 11.1% (OR = 1.111; 95% CI: 1.008–1.225; *p* = 0.034), suggesting a possible link between maternal psychological stress and fetal development. The presence of multiple abnormalities within the same pregnancy was also a significant predictor (OR = 6.382; 95% CI: 1.593–25.558; *p* = 0.009), emphasizing the compounded risk associated with co-occurring anomalies (Table 6).

Overall, the model highlights maternal age, a prior history of congenital anomalies, heightened anxiety levels, and the presence of multiple abnormalities as key risk factors for morphological malformations in this cohort.

+ Factors Associated with Congenital Abnormalities

This section presents the bivariate analyses conducted to assess factors associated with congenital abnormalities. The overall prevalence of congenital abnormalities, including both growth and morphological types, was 13.14%.

+ Congenital Abnormalities and Demographics

A significant association was found between maternal age and the presence of congenital abnormalities. The highest prevalence was observed in the 31–40-year age group (53.6%), with a *p*-value of 0.014. Additionally, the mean age of women with congenital abnormalities (30.96 years) was significantly higher than that of women without abnormalities (28.48 years, *p* = 0.003), indicating maternal age as a potential risk factor.

Marital status was also significantly associated with the occurrence of abnormalities, with a lower prevalence among single women compared to married or divorced women (*p* = 0.001). In contrast, educational level—categorized as “Secondary and less” versus “University and more”—did not show a significant association (*p* = 0.648).

Furthermore, significant associations were observed between congenital abnormalities and the participants’ governorate, residence, and type of residence, suggesting that both biological (e.g., age) and socio-environmental factors (e.g., marital status and living conditions) may influence the risk. However, variables such as nationality, religion, education level, occupation, monthly income, physical activity level, and blood type did not exhibit significant associations.

+ Congenital Abnormalities, Lifestyle Habits, and Comorbidities

Smoking status was not significantly associated with congenital abnormalities (*p* = 0.444). Among smokers, the average number of cigarettes smoked per day was lower in those with abnormalities (4.67) compared to those without (10.57), although the difference was not statistically significant (*p* = 0.111). Similarly, the number of shisha sessions per week (*p* = 0.331) and the duration of smoking (*p* = 0.247) were not significantly different between the groups.

However, hypertension showed a significant association with congenital abnormalities (*p* = 0.028), with a higher prevalence observed among women with abnormalities (3.6%) compared to those without (0.5%) (Table 7).

+ Congenital Abnormalities and Gyneco-Obstetrical Factors

A significant association was observed between the history of stillbirths and the presence of congenital abnormalities (*p* = 0.044). Although the mean number of stillbirths was higher in the group with abnormalities (1.33) than in those without (1.07), this difference did not reach statistical significance (*p* = 0.147).

Both gravidity and parity were significantly associated with congenital abnormalities (*p* < 0.001 for both). Women with abnormalities had higher mean gravidity (3.95 vs. 2.88) and parity (2.93 vs. 2.16), suggesting that an increased number of pregnancies and births may contribute to the risk.

In contrast, gestational age did not show a significant association with congenital abnormalities (*p* = 0.393), with mean values of 23.39 weeks and 23.74 weeks for the affected and unaffected groups, respectively (Table 7).

+ Congenital Abnormalities and Anxiety

While the mean GAD score was higher among women with congenital abnormalities (12.34 ± 4.73) compared to those without (10.74 ± 6.58), the difference did not reach statistical significance (*p* = 0.080). This lack of significance likely reflects a non-linear association, where the overall continuous increase in anxiety score may not accurately capture threshold effects associated with the presence of congenital abnormalities.

While the mean GAD score was higher among women with congenital abnormalities (12.34 ± 4.73) compared to those without (10.74 ± 6.58), this difference did not reach statistical significance (*p* = 0.080). However, when GAD scores were analyzed categorically, a significant association was observed between congenital abnormalities and moderate levels of maternal anxiety (GAD 10–14) (*p* = 0.002), suggesting that intermediate anxiety levels may be particularly relevant.

**Figure 3:**
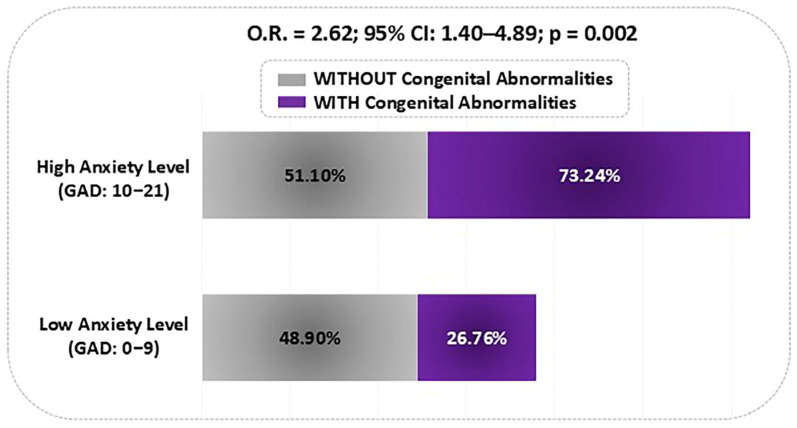
Anxiety level stratification among pregnant women in relation to fetal congenital abnormalities. Proportion of fetal congenital abnormalities among pregnant women categorized by maternal anxiety level. Anxiety was dichotomized into “Low” (GAD 0–9) and “High” (GAD 10–21) to demonstrate the association between elevated anxiety and the occurrence of congenital abnormalities. GAD: Generalized Anxiety Disorder; O.R.: odds ratio; CI: confidence interval.

To further support this association, a simplified dichotomous analysis was conducted, categorizing anxiety as “Low” (GAD 0–9) and “High” (GAD 10–21). This approach revealed that women with high anxiety had significantly higher odds of having fetuses with congenital abnormalities (OR = 2.62; 95% CI: 1.40–4.89; *p* = 0.002), reinforcing the clinical importance of maternal mental health in prenatal risk assessment (Figure 3).

+ Congenital Abnormalities and Other Factors

Amniotic fluid abnormalities (such as oligohydramnios, polyhydramnios, and anhydramnios) were significantly more frequent among cases with congenital abnormalities (7.1%) than those without (1.6%), with a *p*-value of 0.031. This suggests a notable association between amniotic fluid disorders and congenital abnormalities.

However, no significant associations were found regarding intra-uterine treatment (*p* = 1.000) or referrals to tertiary centers (*p* = 0.246), indicating that these interventions or care pathways do not appear to influence the likelihood of congenital abnormalities (Table 8).

+ Binary Logistic Analysis of Risk Factors for Congenital Abnormalities

Binary logistic regression analysis identified several significant predictors of congenital abnormalities (Table 9).

Parity was found to be a statistically significant risk factor, with each additional childbirth increasing the odds of having a child with a congenital abnormality by 32.8% (OR = 1.328; 95% CI: 1.077–1.637; *p* = 0.008). Moreover, the presence of more than one abnormality in a fetus markedly increased the likelihood of congenital abnormalities by more than 10 times (OR = 10.373; 95% CI: 3.432–31.357; *p* < 0.001), making it the strongest predictor in the model. A previous history of a congenital anomaly was also a robust predictor, increasing the odds more than threefold (OR = 3.540; 95% CI: 1.465–8.556; *p* = 0.005) and suggesting a potential genetic or persistent environmental influence. Additionally, obstetric complications—such as a thin lower uterine segment, isthmocele, preterm contractions, uterine artery notch, or ovarian cysts—were associated with a nearly threefold increase in the risk (OR = 2.872; 95% CI: 1.314–6.275; *p* = 0.008) (Table 9).

These findings emphasize the multifactorial nature of congenital abnormalities and highlight the value of comprehensive prenatal risk assessment and targeted monitoring strategies for high-risk pregnancies.

## 4. Discussion

This study identified the clinically relevant prevalence of congenital abnormalities—categorized as growth abnormalities and morphological malformations—as screened through second-trimester prenatal ultrasound (Voluson S6 ultrasound system). The findings underscore the utility of ultrasound as an essential diagnostic tool for early detection and monitoring. Key maternal and obstetric risk factors, including advanced maternal age, hypertension, and moderate levels of anxiety, were significantly associated with abnormal fetal outcomes. These associations highlight the need for integrated prenatal risk assessment to guide timely intervention.

The studied cohort was clinically diverse, with notable exposures including smoking (25.6%) and alcohol consumption (11.7%) during pregnancy, and relatively low rates of hypertension (0.9%) and diabetes (1.6%). A mean GAD-7 score of 10.95/21 and a 9.6% maternal history of congenital anomalies further illustrate the complexity of maternal risk factors in this population.

The observed rates—8.5% for growth abnormalities and 10.1% for morphological malformations—were higher than those reported in similar hospital-based studies from Pakistan [5], Barbados [6], Ethiopia [10], and Nepal [27], but lower than the 29% observed in Tanzania [8]. The prevalence in our study was also lower than that reported in a Lebanese monocentric study by Snaifer et al. [23], which focused on a high-risk refugee population with elevated familial risk. These international variations likely reflect differences in population risk profiles, antenatal care quality, access to screening, and study methodologies.

### 4.1. Growth Abnormalities

Intrauterine growth restriction (IUGR) was the most frequently observed growth abnormality, reinforcing the key role of ultrasound in monitoring both structural and chromosomal anomalies such as a single umbilical artery and down syndrome [28]. Multiple fetal abnormalities were identified in 5.6% of cases, underscoring the complexity of co-existing congenital conditions. Invasive procedures were required in 21.8% of cases, with 17.8% requiring prenatal follow-up and 8.9% postpartum follow-up. These findings highlight the necessity of coordinated antenatal and postnatal care to monitor and manage growth-related complications.

Our findings generally align with the international literature, though notable differences emerge. The significant association between residence type and growth abnormalities (*p* = 0.028), particularly the lower rates among apartment dwellers, is consistent with Appleton et al. (2021) [29] and Workalemahu et al. (2018) [30], who found that favorable environmental and neighborhood conditions are linked to improved fetal outcomes. These parallels likely reflect the protective effect of better living conditions, environmental exposures, and access to health-promoting resources.

The strong link we observed between maternal hypertension and growth abnormalities (*p* = 0.003) agrees with previous reports by Seely & Ecker (2014) [31] and Kooiman et al. (2020) [32], both of whom demonstrated that hypertensive disorders compromise placental blood flow and fetal nutrition, resulting in restricted growth. This concordance suggests that vascular-related pathophysiology remains a key contributor across diverse populations.

Regarding parity, our findings showed a significant association, with higher abnormality rates among multiparous women. This is in line with Al-Farsi et al. (2012) [33], who noted that high parity may lead to cumulative obstetric stress or nutritional depletion, affecting fetal development. However, this contrasts with the results of Kozuki et al. (2013) [34], who reported higher risks in nulliparous women. The divergence may be explained by differences in maternal age distributions, socioeconomic status, and parity-related health behaviors across the studied populations.

The significant association we found between a prior history of congenital anomalies and current growth abnormalities is consistent with findings by Ameen et al. (2018) [35], which point to a genetic or familial predisposition. This emphasizes the relevance of maternal and family medical history in predicting fetal risk.

Obstetric problems were significantly associated with growth abnormalities in our study, echoing the findings of Dapkekar et al. (2023) [36], who identified a range of maternal complications contributing to IUGR. However, this diverges from the conceptual framework proposed by Mayer & Joseph (2013) [26], who cautioned that defining fetal growth abnormalities requires precise criteria and contextual interpretation. The disagreement may be methodological, reflecting differing diagnostic approaches or study populations.

Finally, the strong association between multiple abnormalities and growth restriction (*p* < 0.001) aligns with the comprehensive review by Jagtap et al. (2023) [37], who described the physiological stress and overlapping etiologies involved in coexisting anomalies. This supports the idea that compounded maternal–fetal risk factors intensify the severity and complexity of fetal growth disorders.

Together, these comparative interpretations reveal how methodological differences, population-specific characteristics, and diagnostic criteria can account for both congruence and divergence between our results and those of prior studies. They also highlight the multifactorial etiology of growth abnormalities and the importance of individualized risk stratification in prenatal care.

### 4.2. Morphological Malformations

In parallel with the analysis of growth abnormalities, this study investigated the factors associated with morphological malformations, defined as structural anomalies affecting fetal organ systems. These malformations were found in 10.1% of the study population, underscoring the burden of structural congenital conditions detectable through routine obstetric ultrasound (Voluson S6 ultrasound system).

Advanced maternal age was strongly associated with morphological malformations (*p* < 0.001). This aligns with Kokorudz et al. (2022) [38], who found that advanced maternal age adversely affects embryonic development, especially in brain tissues. Our findings are consistent with their conclusion that maternal age is a key determinant of developmental vulnerability. However, the magnitude of the association in our sample may be accentuated by the specific age distribution of our study population, which included a relatively high proportion of mothers above 35 years of age.

A history of abortion was also significantly associated with morphological malformations. This finding corroborates the results of Sun et al. (2022) [39] and Visconti et al. (2020) [40], both of whom reported increased risks of congenital anomalies in pregnancies following prior miscarriage or abortion. Sun et al. (2022) [39] focused on assisted reproductive technologies and hypothesized a shared underlying etiology (e.g., maternal comorbidities or chromosomal anomalies), while Visconti et al. suggested that recurrent miscarriage and malformations may reflect neglected causal links. The alignment between our results and theirs may, therefore, stem from similar biological and reproductive risk profiles in our study population.

Previous stillbirths were also significantly associated with morphological malformations in our study. This supports the findings of Lean et al. (2017) [41], who attributed increased fetal risk to placental dysfunction, particularly in older women. However, our results diverge from those of Burton and Jauniaux (2018) [42], who found no direct link between previous stillbirths and subsequent malformations. This discrepancy may arise from differences in the studied outcomes—our study focused on structural anomalies while Burton et al. explored growth restriction as a stillbirth mechanism—or from different methodological approaches and population characteristics.

Both high gravidity and high parity were significantly associated with increased risks of malformations (*p* < 0.001 for both). This is in line with the findings of Al-Farsi et al. (2012) [33], who linked high parity to adverse fetal outcomes, possibly due to cumulative physiological burden or depleted maternal reserves. In contrast, Hinkle et al. (2014) [43] did not report a significant relationship between parity and fetal growth, though their focus was on birthweight rather than congenital abnormalities. This divergence may reflect differences in outcome measures, as well as in controlling for confounding factors such as maternal age and socioeconomic status.

A particularly notable finding in our study is the strong association between maternal anxiety and morphological malformations (*p* < 0.001). This aligns with several studies, including De Asis-Cruz et al. (2020) [44], Buss et al. (2010) [45], Wu et al. (2022) [46], and Allison et al. (2011) [47], all of which reported that elevated maternal stress or anxiety during pregnancy can impair fetal brain structure, organogenesis, and neurodevelopment. The mechanisms proposed in these studies include alterations in cortisol levels, placental function, and fetal brain connectivity. Our results may reflect the added impact of chronic stress within the specific context of healthcare instability in Lebanon, as previously described by Fleifel and Abi Farraj (2022) [20].

Lastly, the presence of multiple abnormalities was significantly associated with morphological malformations (*p* < 0.001), echoing Verma (2021) [48], who emphasized that the detection of multiple minor anomalies may signal the presence of more serious structural defects. This supports a syndromic interpretation, in which co-occurring anomalies may share genetic or developmental origins.

Overall, our results align with multiple prior findings, while points of divergence may be explained by differences in outcome definitions (e.g., growth vs. structural anomalies), population characteristics (e.g., maternal age, mental health burden), or contextual healthcare variables (e.g., access to prenatal screening and follow-up). This synthesis reinforces the multifactorial etiology of morphological malformations and the value of comprehensive, context-sensitive screening strategies.

### 4.3. Congenital Abnormalities

This section integrates both growth abnormalities and morphological malformations under the broader category of congenital abnormalities, revealing an overall prevalence of 13.14% in the studied population. This notable rate highlights the significant burden of fetal anomalies and underscores the importance of prenatal ultrasound screening in their early detection and management.

When contextualized within the international literature, the prevalence reported in this study is lower than that observed by Mashuda et al. (2014) in Tanzania (29%) [8], yet higher than those reported in several other countries, including Pakistan (Gillani et al. (2011): 4.24%) [5], Barbados (Singh et al. (2014): 0.6%) [6], Egypt (El Koumi et al. (2013): 1.85%) [49], and Ethiopia (Taye et al. (2019): 2.5%) [10]. These discrepancies may be attributed to a variety of demographic, genetic, environmental, and healthcare-related factors that vary significantly across populations. For instance, maternal age and parity—both associated with fetal anomaly risk—have been shown to influence prevalence rates [34]. Similarly, consanguinity, more common in some Middle Eastern and South Asian populations, is an established risk factor for congenital abnormalities [13]. Healthcare access and preventive care practices also play a critical role. In Tanzania, limited antenatal care and inconsistent folic acid supplementation were linked to higher anomaly rates [8], while in Pakistan, the importance of periconceptional folic acid intake is emphasized as a preventive strategy [5]. In Lebanon, the higher national prevalence reported by Snaifer et al. (2021) [23] could be explained by the characteristics of their study sample, which consisted primarily of refugees with high rates of positive personal and family history of congenital abnormalities, with limited healthcare access, poor antenatal follow-up, and a higher genetic predisposition due to familial clustering and consanguinity. Internationally, recent data from Brazil revealed a strong association between lower maternal education and higher prevalence of major birth defects, emphasizing the impact of socioeconomic factors on anomaly risk [50]. Likewise, Chinese surveillance studies found urban areas to have higher reported rates of congenital anomalies compared to rural areas, potentially due to better diagnostic resources and more consistent prenatal screening [51]. Together, these findings underscore the multifactorial nature of congenital anomaly prevalence and reinforce the need for context-sensitive public health strategies—improving access to maternal education, folic acid supplementation, antenatal care, and prenatal diagnostics tailored to each population’s specific risk profile.

Further analysis using binary logistic regression identified several significant predictors of congenital abnormalities. Specifically, higher parity (*p* = 0.008), the presence of multiple abnormalities (*p* < 0.001), a history of congenital abnormalities (*p* = 0.005), and the occurrence of specific obstetric complications (*p* = 0.008) were all significantly associated with increased risk. These findings support the multifactorial etiology of congenital abnormalities and generally align with previously published studies.

For example, Mashuda et al. (2014) [8] reported that advanced maternal age, inadequate antenatal care, and absence of folic acid supplementation were associated with increased congenital anomaly risk in Tanzania. While our study did not find maternal age to be statistically significant in the final model, the association with parity and obstetric complications is consistent with the role of maternal reproductive history in shaping fetal outcomes. Differences may stem from population-level variations in healthcare access and maternal age distribution.

Likewise, Al-Dewik et al. (2023) [13] identified consanguinity, positive family history, and male infant sex as predictors of congenital anomalies in a large Qatari cohort. Our study corroborates the association with family history, though infant sex and consanguinity were not significantly correlated in our dataset—possibly reflecting different population structures, a smaller sample size, or underreporting of consanguineous relationships in our setting.

Sarkar et al. (2013) [52], in a hospital-based Indian study, found the delivery mode, preterm birth, and maternal BMI to be important predictors. While these variables were not significantly associated in our final analysis, the presence of multiple anomalies and obstetric complications—factors likely interlinked with adverse delivery outcomes—emerged as strong predictors. Such divergence could be attributed to differences in study design, diagnostic methods, sample characteristics, or the broader health system infrastructure in which each study was conducted.

Together, these comparisons emphasize that while there is convergence on some predictors, contextual and methodological differences may account for discrepancies in findings across studies. Our results contribute additional nuance by integrating ultrasound-based screening data and considering both clinical and obstetric variables in the risk model.

In addition to biomedical and obstetric factors, maternal psychological well-being emerged as an important consideration. A significant association was found between high levels of maternal anxiety and the presence of congenital abnormalities. This result is consistent with prior research showing that elevated prenatal anxiety can affect fetal brain development and is linked to adverse cognitive, structural, and emotional outcomes [44,45,46,47]. These findings further support the integration of mental health screening into prenatal care protocols.

Our findings underscore the importance of comprehensive prenatal screening and early risk assessment, enabling timely and targeted interventions. Variations in predictors across studies likely reflect differences in population characteristics and healthcare access. By incorporating both clinical and psychological factors, this study adds nuanced insights to existing knowledge on congenital abnormality risk.

**Figure 4:**
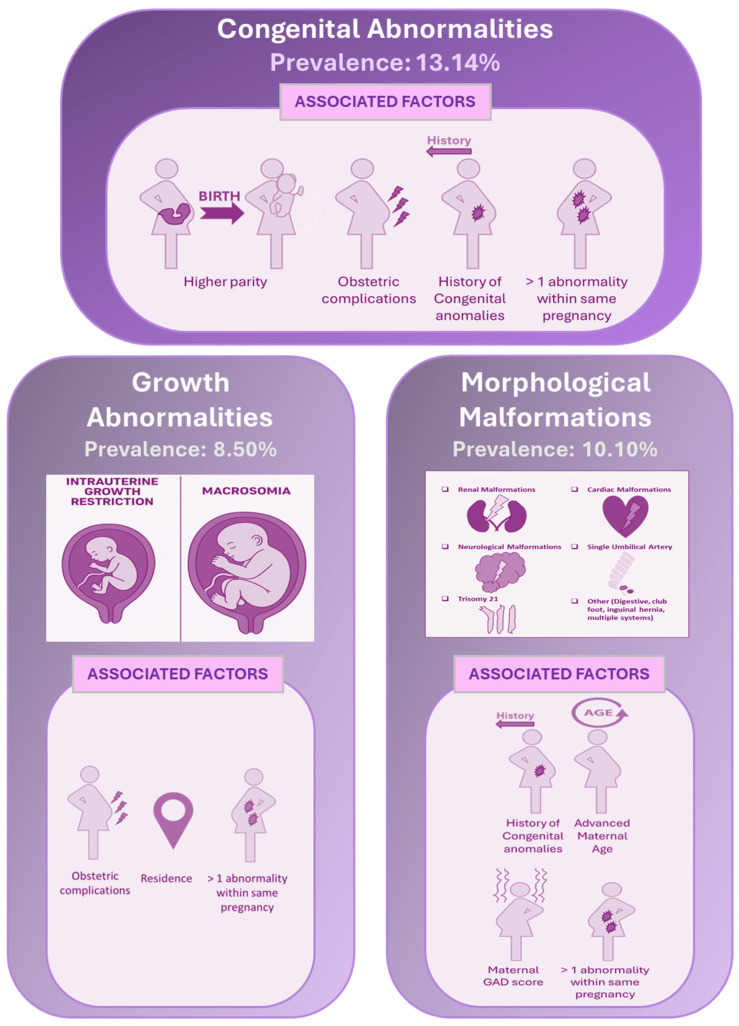
A summary of the prevalence and associated factors (based on binary logistic regression) of congenital abnormalities, including both growth abnormalities and morphological malformations.

These associations are summarized in Figure 4, which presents the prevalence and significant factors associated with growth abnormalities, morphological malformations, and congenital abnormalities (combined), as identified through binary logistic regression.

### 4.4. Limitations

First, this study was conducted as a retrospective observational analysis supplemented by follow-up interviews. Clinical and ultrasound data were obtained from medical records, while missing sociodemographic, lifestyle, and obstetric information was retrospectively collected through structured phone interviews. This dual-source approach was essential to complete data gaps; however, it introduced the potential for time-related inconsistencies and recall bias, particularly in participants’ recollection of personal exposures or behaviors during the second trimester. Although interviewers were trained and participant responses were anchored to the time of ultrasound, retrospective self-reporting inherently carries the risk of memory distortion.

Second, despite efforts to control for confounding variables through multivariate analysis, residual confounding remains a possibility. Some relevant factors may have been unmeasured, incompletely documented in clinical records, or insufficiently adjusted for in the analytical models.

Third, the cross-sectional nature of this study limits any inference of causality. While associations between variables and congenital abnormalities were explored, the temporal sequence of events cannot be determined, and long-term outcomes could not be assessed.

Fourth, selection bias may have influenced the findings. While the sample was drawn from five Order of Malta medical centers selected for their geographic and socioeconomic diversity—and the study population included residents from all eight Lebanese governorates—the sample may not fully capture the heterogeneity of the national population of pregnant women in Lebanon. Women who access services at these centers may differ from those who do not in terms of healthcare-seeking behavior, socioeconomic status, or geographic accessibility. Consequently, some subpopulations—such as women in remote areas, those receiving private prenatal care, or individuals with limited access to ultrasound—may be underrepresented. This underrepresentation could influence the observed prevalence and patterns of congenital abnormalities and may limit the generalizability of our results to the wider Lebanese population.

Lastly, while this study focused on congenital abnormalities detected via second-trimester prenatal ultrasound, the absence of detailed sonographic parameters—such as standardized protocols, gestational age at the time of the scan, and diagnostic thresholds—limits a more granular interpretation of the identified malformations and their clinical relevance.

### 4.5. Perspectives

This study provides valuable insights into the prevalence and determinants of congenital abnormalities among pregnant women in Lebanon, with a focus on both growth abnormalities and morphological malformations. By integrating clinical records with structured interviews, this study offers a comprehensive understanding of risk factors and screening practices in a resource-constrained setting.

From a clinical standpoint, the findings emphasize the importance of strengthening prenatal ultrasound screening, particularly during the second trimester. Early identification of abnormalities enables timely counseling, referral, and intervention. In resource-limited settings such as Lebanon, this requires investment in affordable and portable ultrasound technologies, as well as capacity building for non-specialist healthcare providers—especially trained midwives and general practitioners—to expand the reach of basic fetal anomaly detection services.

From a public health and health systems perspective, our results support the development of nationally standardized prenatal screening protocols. These should prioritize the following:

Early registration of pregnancies; risk-based stratification of cases; structured follow-up for high-risk women; and integration of maternal mental health support, as anxiety was shown to correlate with fetal outcomes.

Health education campaigns should be tailored to address modifiable behavioral risk factors such as smoking and alcohol use during pregnancy, which remain prevalent. Moreover, pediatricians and neonatologists should be closely involved in prenatal care planning, especially in cases where abnormalities are suspected, to ensure smooth postnatal transition and continuity of care. Healthcare administrators also play a vital role in facilitating interdisciplinary coordination, resource allocation, and data-driven planning, particularly through the implementation of national registries for congenital anomalies, which are currently lacking in Lebanon.

These coordinated efforts are essential to improving both maternal and neonatal health outcomes, particularly in low- and middle-income countries.

## 5. Conclusions

This study identified an overall prevalence of congenital abnormalities of 13.14%, with 8.5% presenting as growth abnormalities and 10.1% as morphological malformations. Several maternal and clinical risk factors—including parity, obstetric complications, maternal age, anxiety, and a prior history of anomalies—were significantly associated with adverse fetal outcomes.

Prenatal ultrasound screening, when systematically implemented, proved to be an effective and non-invasive tool for early detection. However, its impact depends heavily on timely access, provider training, and integrated follow-up protocols—elements that require strengthening in the Lebanese context.

To reduce the burden of congenital anomalies and improve perinatal outcomes, comprehensive prenatal care should include not only routine ultrasound assessments but also psychosocial support, health education, and multidisciplinary management. This study’s findings offer concrete guidance for clinicians, pediatric care providers, and healthcare policymakers working to optimize maternal–fetal health in resource-limited environments.

## Figures and Tables

**Table 1:** Characteristics of the population of N = 426 pregnant women. Columns highlighted in purple indicate the variables analyzed in this table, to facilitate visual identification.

		Frequency	Percentage
Age	<18 years	5	1.2
	18–30 years	252	59.2
	31–40 years	155	36.4
	41–50 years	14	3.3
Age	Analyzed N	426	
	Mean (SD)	28.80 (5.93)	
	Min–Max	14.00–49.00	
Nationality	Lebanese	313	73.5
	Syrian	111	26.1
	Others	2	0.5
Religion	Christian	133	31.2
	Muslim	273	64.1
	Druze	19	4.5
	Does not want to answer	1	0.2
Education level	Illiterate	12	2.8
	Primary	88	20.7
	Complementary	75	17.6
	Secondary	64	15.0
	University	176	41.3
	Higher education	11	2.6
Marital Status	Single	3	0.7
	Married	421	98.8
	Divorced	2	0.5
Occupation	Unemployed	323	75.8
	Employed	103	24.2
Monthly income in USD	<50	34	8.0
	50–100	53	12.4
	100–150	101	23.7
	150–200	88	20.7
	200–250	34	8.0
	250–500	76	17.8
	500–750	28	6.6
	>750	12	2.8
Governorate	Beirut	30	7.0
	Mount Lebanon	22	5.2
	Bekaa	78	18.3
	Baalback-Hermel	13	3.1
	North Lebanon	38	8.9
	Akkar	212	49.8
	South Lebanon	30	7.0
	Nabatiyeh	3	0.7
Residence	Alone	213	50.0
	With others	213	50.0
How many rooms in your residence (excluding kitchen and bathroom)	Analyzed N	426	
	Mean (SD)	2.81 (1.06)	
	Min–Max	1.00–6.00	
Residence type	Apartment	343	80.5
	Shelter	26	6.1
	Others	57	13.4
Activity level	Sedentary	114	26.8
	Lightly active	138	32.4
	Moderately active	139	32.6
	Very active	35	8.2
Blood type	A+	150	35.2
	A−	26	6.1
	B+	45	10.6
	B−	5	1.2
	O+	139	32.6
	O−	17	4.0
	AB+	22	5.2
	AB−	4	0.9
	Unknown	18	4.2
Weight (in kg)	Analyzed N	412	
	Mean (SD)	67.51 (14.74)	
	Min–Max	40.00–140.00	
Height (in cm)	Analyzed N	412	
	Mean (SD)	161.70 (6.64)	
	Min–Max	140.00–183.00	
BMI	Analyzed N	412	
	Mean (SD)	25.80 (5.25)	
	Min–Max	15.63–50.81	
Smoker	No	317	74.4
	Yes	109	25.6
If yes, on average, how many cigarettes do you smoke per day	Analyzed N	31	
	Mean (SD)	10.00 (6.08)	
	Min–Max	1.00–20.00	
If yes, on average, how many shishas do you smoke per week	Analyzed N	70	
	Mean (SD)	5.90 (4.20)	
	Min–Max	0.40–20.00	
If yes, how many years have you been smoking for	Analyzed N	97	
	Mean (SD)	7.68 (4.55)	
	Min–Max	1.00–20.00	
Alcohol consumption	Never	376	88.3
	1 time per month or less	29	6.8
	2–4 times per month	20	4.7
	4 times or more per week	1	0.2
Alcohol consumption	No	376	88.3
	Yes	50	11.7
Hypertension	No	422	99.1
	Yes	4	0.9
Diabetes	No	419	98.4
	Yes	7	1.6
Fetal Phenotype	Male	219	51.4
	Female	192	45.1
	Twins	10	2.3
	Triplet	2	0.5
	Unknown	3	0.7
Abortion	No	260	61.0
	Yes	166	39.0
If yes, specify how many abortions the patient had	Analyzed N	164	
	Mean (SD)	1.62 (0.96)	
	Min–Max	1.00–7.00	
History of stillbirths	No	404	94.8
	Yes	22	5.2
If yes, specify how many stillbirths the patient had	Analyzed N	20	
	Mean (SD)	1.15 (0.37)	
	Min–Max	1.00–2.00	
Gestational Age (weeks)	Analyzed N	426	
	Mean (SD)	23.69 (2.77)	
	Min–Max	14.40–30.60	
Gravidity	Analyzed N	424	
	Mean (SD)	3.02 (2.09)	
	Min–Max	1.00–22.00	
Parity	Analyzed N	416	
	Mean (SD)	2.26 (1.35)	
	Min–Max	0.00–9.00	
Consanguinity	No	338	79.3
	Yes	88	20.7
COVID-19 vaccine	No	220	51.6
	Yes	206	48.4
Infertility problem	No	385	90.4
	Yes	41	9.6
Exposure to infections or radiation during the first trimester	No	317	74.4
	Yes	109	25.6

**Table 2:** Association of lifestyle habits and comorbidities and gyneco-obstetrical problems with growth abnormalities. Columns highlighted in purple indicate the variables analyzed in this table, to facilitate visual identification. *p*-values presented in bold denote statistical significance.

		Growth Abnormalities (Macrosomia, IUGR, Growth vs. Wrong Dating)		Total	*p*-Value
		No	Yes		
Smoker	No	289	28	317	0.629
		74.1%	77.8%	74.4%	
	Yes	101	8	109	
		25.9%	22.2%	25.6%	
If yes, on average, how many cigarettes do you smoke per day	Analyzed N	29	2	31	0.119
	Mean (SD)	10.45 (6.03)	3.50 (0.71)	10.00 (6.08)	
	Min–Max	1.0–20.0	3.0–4.0	1.0–20.0	
If yes, on average, how many shisha do you smoke per week	Analyzed N	64	6	70	0.656
	Mean (SD)	5.97 (4.13)	5.17 (5.23)	5.90 (4.20)	
	Min–Max	0.4–20.0	1.0–14.0	0.4–20.0	
If yes, how many years have you been smoking for	Analyzed N	90	7	97	0.428
	Mean (SD)	7.58 (4.34)	9.00 (6.98)	7.68 (4.55)	
	Min–Max	1.0–20.0	1.0–20.0	1.0–20.0	
Alcohol Consumption	Never	344	32	376	0.911
		88.2%	88.9%	88.3%	
	1 time per month or less	26	3	29	
		6.7%	8.3%	6.8%	
	2–4 times per month	19	1	20	
		4.9%	2.8%	4.7%	
	4 times or more per week	1	0	1	
		0.3%	0.0%	0.2%	
Alcohol Consumption	No	344	32	376	0.903
		88.2%	88.9%	88.3%	
	Yes	46	4	50	
		11.8%	11.1%	11.7%	
Weight (in kg)	Analyzed N	376	36	412	0.191
	Mean (SD)	67.22 (14.50)	70.58 (16.96)	67.51 (14.74)	
	Min–Max	40.0–140.0	40.0–110.0	40.0–140.0	
Height (in cm)	Analyzed N	376	36	412	0.378
	Mean (SD)	161.79 (6.63)	160.76 (6.79)	161.70 (6.64)	
	Min–Max	140.0–177.0	147.0–183.0	140.0–183.0	
BMI	Analyzed N	376	36	412	0.089
	Mean (SD)	25.66 (5.16)	27.22 (6.00)	25.80 (5.25)	
	Min–Max	15.6–50.8	17.8–42.2	15.6–50.8	
Hypertension	No	388	34	422	**0.003**
		99.5%	94.4%	99.1%	
	Yes	2	2	4	
		0.5%	5.6%	0.9%	
Diabetes	No	383	36	419	0.418
		98.2%	100.0%	98.4%	
	Yes	7	0	7	
		1.8%	0.0%	1.6%	
Fetal Phenotype	Male	203	16	219	0.253
		52.1%	44.4%	51.4%	
	Female	174	18	192	
		44.6%	50.0%	45.1%	
	Twins	9	1	10	
		2.3%	2.8%	2.3%	
	Triplet	1	1	2	
		0.3%	2.8%	0.5%	
	Unknown	3	0	3	
		0.8%	0.0%	0.7%	
Gyneco-Obstetrical factors					
Abortion	No	239	21	260	0.729
		61.3%	58.3%	61.0%	
	Yes	151	15	166	
		38.7%	41.7%	39.0%	
If yes, specify how many abortions the patient had	Analyzed N	149	15	164	0.709
	Mean (SD)	1.63 (0.98)	1.53 (0.83)	1.62 (0.96)	
	Min–Max	1.0–7.0	1.0–4.0	1.0–7.0	
History of stillbirths	No	370	34	404	0.912
		94.9%	94.4%	94.8%	
	Yes	20	2	22	
		5.1%	5.6%	5.2%	
If yes, specify how many stillbirths the patient had	Analyzed N	18	2	20	0.160
	Mean (SD)	1.11 (0.32)	1.50 (0.71)	1.15 (0.37)	
	Min–Max	1.0–2.0	1.0–2.0	1.0–2.0	
Gestational Age (weeks)	Analyzed N	390	36	426	0.663
	Mean (SD)	23.67 (2.78)	23.88 (2.80)	23.69 (2.77)	
	Min–Max	14.4–30.6	15.9–28.6	14.4–30.6	
Gravidity	Analyzed N	388	36	424	0.177
	Mean (SD)	2.98 (2.11)	3.47 (1.89)	3.02 (2.09)	
	Min–Max	1.0–22.0	1.0–7.0	1.0–22.0	
Parity	Analyzed N	380	36	416	**0.044**
	Mean (SD)	2.22 (1.34)	2.69 (1.45)	2.26 (1.35)	
	Min–Max	0.0–9.0	1.0–7.0	0.0–9.0	
Previous history of a congenital anomaly	No	353	28	381	**0.017**
		90.5%	77.8%	89.4%	
	Yes	37	8	45	
		9.5%	22.2%	10.6%	
Consanguinity	No	311	27	338	0.501
		79.7%	75.0%	79.3%	
	Yes	79	9	88	
		20.3%	25.0%	20.7%	
COVID-19 vaccine	No	204	16	220	0.366
		52.3%	44.4%	51.6%	
	Yes	186	20	206	
		47.7%	55.6%	48.4%	
Infertility problem	No	354	31	385	0.372
		90.8%	86.1%	90.4%	
	Yes	36	5	41	
		9.2%	13.9%	9.6%	
Exposure to infections or radiations during the first trimester	No	293	24	317	0.266
		75.1%	66.7%	74.4%	
	Yes	97	12	109	
		24.9%	33.3%	25.6%	

**Table 3:** Binary logistic analysis for the risk factors of growth abnormalities. Columns highlighted in purple indicate the variables analyzed in this table, to facilitate visual identification. *p*-values presented in bold denote statistical significance.

	B	S.E.	Sig.	Exp(B)	95% C.I.for EXP(B)	
					Lower Upper	
Obstetric problems (thin lower uterine segment, isthmocele, preterm contractions during examination, notch on uterine artery, ovarian cysts)	1.448	0.430	**0.001**	4.254	1.830	9.891
Cases with >1 abnormalities	2.619	0.506	**<0.001**	13.721	5.085	37.022
Constant	−2.907	0.249	**<0.001**	0.055		

Dependent: Growth abnormalities (0: No/1: Yes); variable(s) entered in the model: cases with >1 abnormalities: (0: No/1: Yes); obstetric problems (thin lower uterine segment, isthmocele, preterm contractions during examination, notch on uterine artery, ovarian cysts): (0: No/1: Yes).

**Table 4:** Morphological malformations and gyneco-obstetrical factors. Columns highlighted in purple indicate the variables analyzed in this table, to facilitate visual identification. *p*-values presented in bold denote statistical significance.

		Morphological Malformations		Total	*p*-Value
		No	Yes		
Abortion	No	251	9	260	**0.004**
		62.8%	34.6%	61.0%	
	Yes	149	17	166	
		37.3%	65.4%	39.0%	
If yes, specify how many abortions the patient had	Analyzed N	147	17	164	0.363
	Mean (SD)	1.60 (0.92)	1.82 (1.29)	1.62 (0.96)	
	Min–Max	1.0–7.0	1.0–5.0	1.0–7.0	
History of stillbirths	No	382	22	404	**0.038**
		95.5%	84.6%	94.8%	
	Yes	18	4	22	
		4.5%	15.4%	5.2%	
If yes, specify how many stillbirths the patient had	Analyzed N	16	4	20	0.556
	Mean (SD)	1.13 (0.34)	1.25 (0.50)	1.15 (0.37)	
	Min–Max	1.0–2.0	1.0–2.0	1.0–2.0	
Gestational Age (weeks)	Analyzed N	400	26	426	**0.041**
	Mean (SD)	23.76 (2.74)	22.61 (3.12)	23.69 (2.77)	
	Min–Max	14.4–30.4	15.9–30.6	14.4–30.6	
Gravidity	Analyzed N	399	25	424	**<0.001**
	Mean (SD)	2.92 (2.00)	4.68 (2.79)	3.02 (2.09)	
	Min–Max	1.0–22.0	1.0–12.0	1.0–22.0	
Parity	Analyzed N	392	24	416	**<0.001**
	Mean (SD)	2.20 (1.29)	3.29 (1.92)	2.26 (1.35)	
	Min–Max	0.0–9.0	1.0–7.0	0.0–9.0	
Previous history of a congenital anomaly	No	369	12	381	**<0.001**
		92.3%	46.2%	89.4%	
	Yes	31	14	45	
		7.8%	53.8%	10.6%	
Consanguinity	No	319	19	338	0.415
		79.8%	73.1%	79.3%	
	Yes	81	7	88	
		20.3%	26.9%	20.7%	
COVID-19 vaccine	No	205	15	220	0.524
		51.3%	57.7%	51.6%	
	Yes	195	11	206	
		48.8%	42.3%	48.4%	
Infertility problem	No	362	23	385	0.729
		90.5%	88.5%	90.4%	
	Yes	38	3	41	
		9.5%	11.5%	9.6%	
Exposure to infections or radiation during the first trimester	No	300	17	317	0.276
		75.0%	65.4%	74.4%	
	Yes	100	9	109	
		25.0%	34.6%	25.6%	

**Table 5:** Malformation anomalies, GAD, and other factors. Columns highlighted in purple indicate the variables analyzed in this table, to facilitate visual identification. *p*-values presented in bold denote statistical significance.

		Morphological Malformations		Total	*p*-Value
		No	Yes		
Anxiety	Minimal anxiety (GAD 0–4)	63	1	64	**<0.001**
		15.8%	3.8%	15.0%	
	Mild anxiety (GAD 5–9)	131	1	132	
		32.8%	3.8%	31.0%	
	Moderate anxiety (GAD 10–14)	116	16	132	
		29.0%	61.5%	31.0%	
	Severe anxiety (GAD 15–21)	90	8	98	
		22.5%	30.8%	23.0%	
GAD	Analyzed N	400	26	426	**0.013**
	Mean (SD)	10.75 (6.46)	13.96 (4.10)	10.95 (6.39)	
	Min–Max	0.0–21.0	0.0–21.0	0.0–21.0	
Amniotic fluid problems (oligohydramnios, polyhydramnios, anamnios)	No	392	24	416	0.119
		98.0%	92.3%	97.7%	
	Yes	8	2	10	
		2.0%	7.7%	2.3%	
Obstetric problems (thin lower uterine segment, isthmocele, preterm contractions during examination, notch on uterine artery, ovarian cysts)	No	347	23	370	1.000
		86.8%	88.5%	86.9%	
	Yes	53	3	56	
		13.3%	11.5%	13.1%	
Intra-utero treatment	No	397	26	423	1.000
		99.3%	100.0%	99.3%	
	Yes	3	0	3	
		0.8%	0.0%	0.7%	
Invasive procedures	No	309	24	333	0.086
		77.3%	92.3%	78.2%	
	Yes	91	2	93	
		22.8%	7.7%	21.8%	
Referred to tertiary center	No	399	25	424	0.118
		99.8%	96.2%	99.5%	
	Yes	1	1	2	
		0.3%	3.8%	0.5%	
Cases with >1 abnormalities	No	387	15	402	**<0.001**
		96.8%	57.7%	94.4%	
	Yes	13	11	24	
		3.3%	42.3%	5.6%	

**Table 6:** Binary logistic analysis for the risk factors of morphological malformations. Columns highlighted in purple indicate the variables analyzed in this table, to facilitate visual identification. *p*-values presented in bold denote statistical significance.

	B	S.E.	Sig.	Exp(B)	95% C.I. for EXP(B)	
					Lower	Upper
Age	0.132	0.044	**0.003**	1.141	1.046	1.245
Previous history of a congenital anomaly	1.943	0.622	**0.002**	6.982	2.064	23.616
GAD	0.105	0.050	**0.034**	1.111	1.008	1.225
Cases with >1 abnormalities	1.853	0.708	**0.009**	6.382	1.593	25.558
Constant	−8.978	1.628	**0.000**	0.000		

Dependent: Morphological malformations (0: No/1: Yes); variable(s) entered in the model: age (continuous); previous history of a congenital anomaly: (0: No/1: Yes); GAD (continuous); cases with >1 abnormalities: (0: No/1: Yes).

**Table 7:** Congenital abnormalities, gyneco-obstetrical factors, and comorbidities. Columns highlighted in purple indicate the variables analyzed in this table, to facilitate visual identification. *p*-values presented in bold denote statistical significance.

		Congenital Abnormalities		Total	*p*-Value
		No	Yes		
Smoker	No	273	44	317	0.444
		73.8%	78.6%	74.4%	
	Yes	97	12	109	
		26.2%	21.4%	25.6%	
If yes, on average how many cigarettes do you smoke per day	Analyzed N	28	3	31	0.111
	Mean (SD)	10.57 (6.10)	4.67 (2.08)	10.00 (6.08)	
	Min–Max	1.0–20.0	3.0–7.0	1.0–20.0	
If yes, on average how many shisha do you smoke per week	Analyzed N	60	10	70	0.331
	Mean (SD)	6.10 (4.16)	4.70 (4.42)	5.90 (4.20)	
	Min–Max	0.4–20.0	1.0–14.0	0.4–20.0	
If yes, how many years have you been smoking for	Analyzed N	86	11	97	0.247
	Mean (SD)	7.49 (4.22)	9.18 (6.65)	7.68 (4.55)	
	Min–Max	1.0–20.0	1.0–20.0	1.0–20.0	
Alcohol consumption	Never	329	47	376	0.317
		88.9%	83.9%	88.3%	
	1 time per month or less	22	7	29	
		5.9%	12.5%	6.8%	
	2–4 times per month	18	2	20	
		4.9%	3.6%	4.7%	
	4 times or more per week	1	0	1	
		0.3%	0.0%	0.2%	
Alcohol consumption	No	329	47	376	0.280
		88.9%	83.9%	88.3%	
	Yes	41	9	50	
		11.1%	16.1%	11.7%	
Weight (in kg)	Analyzed N	357	55	412	0.470
	Mean (SD)	67.31 (14.68)	68.85 (15.21)	67.51 (14.74)	
	Min–Max	40.0–140.0	40.0–110.0	40.0–140.0	
Height (in cm)	Analyzed N	357	55	412	0.223
	Mean (SD)	161.85 (6.68)	160.68 (6.39)	161.70 (6.64)	
	Min–Max	140.0–177.0	147.0–183.0	140.0–183.0	
BMI	Analyzed N	357	55	412	0.201
	Mean (SD)	25.67 (5.20)	26.64 (5.59)	25.80 (5.25)	
	Min–Max	15.6–50.8	17.8–42.2	15.6–50.8	
Hypertension	No	368	54	422	**0.028**
		99.5%	96.4%	99.1%	
	Yes	2	2	4	
		0.5%	3.6%	0.9%	
Diabetes	No	363	56	419	0.299
		98.1%	100.0%	98.4%	
	Yes	7	0	7	
		1.9%	0.0%	1.6%	
Fetal Phenotype	Male	193	26	219	0.466
		52.2%	46.4%	51.4%	
	Female	164	28	192	
		44.3%	50.0%	45.1%	
	Twins	9	1	10	
		2.4%	1.8%	2.3%	
	Triplet	1	1	2	
		0.3%	1.8%	0.5%	
	Unknown	3	0	3	
		0.8%	0.0%	0.7%	
Abortion	No	232	28	260	0.069
		62.7%	50.0%	61.0%	
	Yes	138	28	166	
		37.3%	50.0%	39.0%	
If yes, specify how many abortions the patient had	Analyzed N	136	28	164	0.578
	Mean (SD)	1.60 (0.92)	1.71 (1.15)	1.62 (0.96)	
	Min–Max	1.0–7.0	1.0–5.0	1.0–7.0	
History of stillbirths	No	354	50	404	**0.044**
		95.7%	89.3%	94.8%	
	Yes	16	6	22	
		4.3%	10.7%	5.2%	
If yes, specify how many stillbirths the patient had	Analyzed N	14	6	20	0.147
	Mean (SD)	1.07 (0.27)	1.33 (0.52)	1.15 (0.37)	
	Min–Max	1.0–2.0	1.0–2.0	1.0–2.0	
Gestational Age (weeks)	Analyzed N	370	56	426	0.393
	Mean (SD)	23.74 (2.75)	23.39 (2.92)	23.69 (2.77)	
	Min–Max	14.4–30.4	15.9–30.6	14.4–30.6	
Gravidity	Analyzed N	369	55	424	**<0.001**
	Mean (SD)	2.88 (2.02)	3.95 (2.36)	3.02 (2.09)	
	Min–Max	1.0–22.0	1.0–12.0	1.0–22.0	
Parity	Analyzed N	362	54	416	**<0.001**
	Mean (SD)	2.16 (1.27)	2.93 (1.69)	2.26 (1.35)	
	Min–Max	0.0–9.0	1.0–7.0	0.0–9.0	
Previous history of a congenital anomaly	No	344	37	381	**<0.001**
		93.0%	66.1%	89.4%	
	Yes	26	19	45	
		7.0%	33.9%	10.6%	
Consanguinity	No	294	44	338	0.878
		79.5%	78.6%	79.3%	
	Yes	76	12	88	
		20.5%	21.4%	20.7%	
COVID-19 vaccine	No	193	27	220	0.582
		52.2%	48.2%	51.6%	
	Yes	177	29	206	
		47.8%	51.8%	48.4%	
Infertility problem	No	336	49	385	0.434
		90.8%	87.5%	90.4%	
	Yes	34	7	41	
		9.2%	12.5%	9.6%	
Exposure to infections or radiation during the first trimester	No	281	36	317	0.062
		75.9%	64.3%	74.4%	
	Yes	89	20	109	
		24.1%	35.7%	25.6%	

**Table 8:** Association of congenital anomalies with other factors. Columns highlighted in purple indicate the variables analyzed in this table, to facilitate visual identification. *p*-values presented in bold denote statistical significance.

		Congenital Abnormalities		Total	*p*-Value
		No	Yes		
Amniotic fluid problems (oligohydramnios, polyhydramnios, anamnios)	No	364	52	416	**0.031**
		98.4%	92.9%	97.7%	
	Yes	6	4	10	
		1.6%	7.1%	2.3%	
Obstetric problems (thin lower uterine segment, isthmocele, preterm contractions during examination, notch on uterine artery, ovarian cysts)	No	326	44	370	**0.049**
		88.1%	78.6%	86.9%	
	Yes	44	12	56	
		11.9%	21.4%	13.1%	
Intra-utero treatment	No	367	56	423	1.000
		99.2%	100.0%	99.3%	
	Yes	3	0	3	
		0.8%	0.0%	0.7%	
Invasive procedures	No	283	50	333	**0.031**
		76.5%	89.3%	78.2%	
	Yes	87	6	93	
		23.5%	10.7%	21.8%	
Referred to tertiary center	No	369	55	424	0.246
		99.7%	98.2%	99.5%	
	Yes	1	1	2	
		0.3%	1.8%	0.5%	
Cases with >1 abnormalities	No	362	40	402	**<0.001**
		97.8%	71.4%	94.4%	
	Yes	8	16	24	
		2.2%	28.6%	5.6%	

**Table 9:** Binary logistic analysis for the risk factors of congenital abnormalities. Columns highlighted in purple indicate the variables analyzed in this table, to facilitate visual identification. *p*-values presented in bold denote statistical significance.

	B	S.E.	Sig.	Exp(B)	95% CI for EXP(B)	
					Lower	Upper
Parity	0.283	0.107	**0.008**	1.328	1.077	1.637
Cases with >1 abnormalities	2.339	0.564	**<** **0.00** **1**	10.373	3.432	31.357
Previous history of a congenital anomaly	1.264	0.450	**0.005**	3.540	1.465	8.556
Obstetric problems (thin lower uterine segment, isthmocele, preterm contractions during examination, notch on uterine artery, ovarian cysts)	1.055	0.399	**0.008**	2.872	1.314	6.275
Constant	−3.229	0.357	**<** **0.00** **1**	0.040		

Dependent: congenital abnormalities (0: No/1: Yes); variable(s) entered in the model: parity (continuous); cases with >1 abnormalities: (0: No/1: Yes); previous history of a congenital anomaly: (0: No/1: Yes); obstetric problems (thin lower uterine segment, isthmocele, preterm contractions during examination, notch on uterine artery, ovarian cysts): (0: No/1: Yes).

## Data Availability

The data presented in this study are available on request from the corresponding author. The data are not publicly available due to their containing information that could compromise the privacy of research participants.

## Abbreviations

BMI: Body Mass Index; FASD: Fetal Alcohol Spectrum Disorders; GAD-7: Generalized Anxiety Disorder; GUD: Genitourinary Defects; LBW: Low Birth Weight; NTD: Neural Tube Defects; NTFD: Neural Tube Fusion Defects; OBGYN: Obstetrics and Gynecology; WHO: World Health Organization.
